# Flat Top Talus: Complication of Ponseti Method or Overcorrection?

**DOI:** 10.7759/cureus.13390

**Published:** 2021-02-17

**Authors:** Shahbaz Khan, Mansoor Ali Khan, Muhammad Amin Chinoy, Sadia Ahmed

**Affiliations:** 1 Orthopaedics and Traumatology, Ziauddin University Hospital Karachi, Karachi, PAK; 2 Orthopaedics, The Indus Hospital, Karachi, PAK; 3 Research, The Indus Hospital, Karachi, PAK

**Keywords:** flat top talus, ponseti, clubfoot, ta tenotomy

## Abstract

Purpose

Deformation of talus in idiopathic clubfoot is a common problem both surgically and after treatment with the Ponseti technique, although the cause of deformation and its clinical impact on the function of the ankle is not yet known. The goal of this research was to evaluate factors leading to talar dome deformation (flat-top talus) after the Ponseti technique

Methods

This was a single-center, cross-sectional study. Fifty patients with virgin idiopathic clubfoot were enrolled from our consecutive series of data from August 2017 to January 2018 from our clubfoot patients who completed their casting and bracing protocol. Weight-bearing lateral X-rays of the ankle were examined in patients to determine the flattening of the talus dome and its correlation with age, sex, BMI, number of casts, and casting period. In these patients, the frequency of tenotomy and its relationship to the flat top talus was also examined.

Results

The study included a total of 50 children, of which 36 (72%) were boys and 14 (28%) were girls. The mean age, height, weight, and BMI of the children were 5.06 ± 0.79 years, 101.6 ± 6.34 cm, and 19.7 ± 1.57 kg, respectively. No significant difference between the normal and flat top talus category was found in age and BMI (p=0.611 and 0.997, respectively). Whereas, relative to normal children, the children who had flat-top talus were on casts for a longer period of time (median: 9 vs. 6 weeks, p=0.026). In addition, a higher proportion of children with more than six casts developed flat-top talus than those with fewer than six casts (69.2% vs. 30.8%, p=0.005). After treatment, a total of 13 (26%) patients developed flat top talus, of which 11 (84.6%) were boys and two (15.4%) were girls (p=0.303). No substantial association between tenotomy and flat top talus (p=0.340) could be identified.

Conclusion

Flat top talus is a complication of improper manipulation specifically correlated with the number of Ponseti casts applied. Maintenance of cast treatment for more than three months may result in flat-top talus with no significant association with tenotomy of the tendoachilles.

## Introduction

Idiopathic congenital clubfoot is a complex and relatively common deformity [1]. Complexity is based on the presence of various deformities in all parts of the foot. Congenital talipes equinovarus (CTEV) varies in severity and stiffness, so not all CTEV feet are similar [2,3]. Pathological anomalies linked to muscles, soft tissues, nerves, and vessels have been identified although the origin of the idiopathic variety is unclear [4].

Management of this complex deformity is by non-surgical and surgical methods. Non-operative treatment has been accepted as a method of choice in recent decades, particularly since the implementation and widespread acceptance of the Ponseti method [5,6]. Kite and French methods [7] led to an increased number of cases treated by manipulation and casting in past but the sequence of manipulation was ineffective as compared to the Ponseti method. The general objective is the same: to achieve complete and permanent correction and optimum foot function with minimal after-effects and morbidity.

Talar dome deformation is seen after surgical and non-surgical treatment of CTEV, but there is no agreement on the factors leading to it to date. Reports demonstrate that the flattening of the talus reduces the dynamic mobility of the ankle [8,9]. There is little evidence of various trends of flattening of the talus dome [10].

The first aim of this study was to define the causes of talus deformation, its relationship with Ponseti casting, and errors related to the method leading to talus flattening. The second aim was to determine whether there is an association of foot correction without tenotomy with the development of talus flattening to negate the hypothesis of the nutcracker effect.

## Materials and methods

The research was carried out using a cross-sectional study design from august 2017 to January 2018 in a single center. After institutional review board (IRB) approval, from the consecutive series enrolled in the clubfoot program at our hospital, a total of 50 patients with virgin idiopathic clubfoot with no previous treatment who completed the casting and bracing regimen for four years were included in the study after getting written consent. Patients who were lost to follow-up during the casting or bracing protocol and those with other conditions like meningomyelocele, arthrogryposis were excluded from the study.

To determine the deformation in the shape of the talus, patients underwent a weight-bearing lateral view of the ankle joint. The patients were accompanied by the researcher (Senior Consultant) to get the true ankle joint lateral image. By placing the child on a step stool to take a standing lateral view, with the lateral border of the foot touching the cassette and rays perpendicular to the foot, X-rays of the ankle joint were taken. Data on current age, age at the time of registration, sex, Pirani scoring deformity, number of Ponseti casts, tenotomy performed or not, ankle dorsiflexion after tenotomy, complications associated with casting, length of casting, and flat-top talus reported as slightly flattened, significantly altered, or flat depending on the sphericity of the Tibiotalar joint using Mose circle method was collected. 

Data was entered and analyzed using SPSS version 21.0 (IBM Corp., Armonk, NY, USA). Mean and standard deviation was computed for all the normally distributed quantitative variables. Median and interquartile ranges were computed for all the non-normally distributed quantitative variables. Frequencies and proportions were computed for all the categorical variables like gender, tenotomy performed, Ponseti cast-related complications, and flat-top talus. Potential confounders like age, BMI, number of Ponseti casts, duration of casting, tenotomy performed or not were controlled through stratification. Post-stratification Chi-square test/Fisher-exact test was applied. P-value<0.05 was considered statistically significant.

## Results

In the study, a total of 50 patients were enrolled. The mean age of the participants in the sample was 5.06 (± 0.79) years, 36 (72%) were males and only 14 (28%) were females. Overall, 17 (34%) of the patients had more than six casts, while 33 (66%) of the patients got less than or equivalent to six casts. 58% of patients underwent a tenotomy procedure and 26% of patients were diagnosed with flat-top talus (FTT). No significant difference was found in the present age and BMI between the normal and flat top talus groups (p=0.611 and 0.997 respectively) (Table 1). Children who were diagnosed with FTT were older relative to average (p=0.044) at the time of registration. In addition, in patients undergoing tenotomy, the mean number of casts applied prior to tenotomy was found to be higher in the FTT group (6.6 ± 5.1) than in the non-FTT group (9.2 ± 3.7), but the findings were statistically insignificant (p=0.10). In addition, the average Pirani induction score in both cohorts was statistically insignificant (p=0.942). In the FTT group patients, the overall number of casts applied was slightly higher (p=0.005) and the bracing length was lower than in the non-FTT group (p=0.058) (Table 2). After treatment, a total of 13 (26%) patients developed flat-top talus, 11 (84.6%) of which were boys and two (15.4%) were girls (p=0.303). No correlation between tenotomy and flat-top talus has been identified (p=0.340) (Table 3). The higher Pirani induction score is found to be correlated with a higher number of casts, thereby increasing FTT development chances.

**Table 1:** Demographical information of Patients (n=50) IQR: interquartile range

Age (years)	
Mean ± SD	5.06 ± 0.79
Height (cm)	
Mean ± SD	101.6 ± 6.34
Weight (kg)	
Mean ± SD	19.7 ± 1.57
BMI	
Mean ± SD	19.30 ± 2.4
Duration of cast (weeks)	
Median (IQR)	7 (6 - 10)
Gender n (%)	
Male	36 (72)
Tenotomy n (%)	29 (58)
Cast n (%)	
More than 6 times	17 (34)
Less than equal to 6 times	33 (66)
Flat top talus n (%)	13 (26)

**Table 2:** Comparison between flat-top talus group and normal group IQR: interquartile range

FTT Absent n=37		FTT Present n=13			
Mean ± SD	Median (IQR)	Mean ± SD	Median (IQR)	P-value	
Present Age in years	5.1 ± 0.8	5 (4 - 6)	5 ± 0.913	5 (4 - 5)	0.611	
Age at the time of induction in years	0.5 ± 0.8	0.2 (0.1-0.5)	1.3 ± 1.3	0.71 (0.2-2.7)	0.044	
Age at the time of induction in days	190.9 ± 276.7	84 (42-178)	469.3 ± 481	259 (69-1004)	0.044	
Number of casts applied	7.4 ± 3.1	6 (6 - 9)	9.75 ± 3.2	9 (7 - 13)	0.026	
Number of casts before tenotomy	6.6 ± 5.1	5 (4-8)	9.2 ± 3.7	8.5 (6.2-12)	0.108	
BMI	19.3 ± 2.3	20 (17.3 - 21.5)	19.3 ± 2.3	19 (17.9 - 20)	0.997	
Duration of bracing in months	119.6 ± 27.4	118.4 (112.3-131.2)	102.6 ± 26.6	109.1 (90.3-118)	0.058	
Average Pirani score at the time of induction	3.5 ± 1.5	3 (2.6-4.8)	3.5 ± 2.0	3.8 (2.2-5.2)	0.942	

**Table 3:** Comparison of FTT and non-FTT groups FTT: flat-top talus

	Flat-top talus		Total n=50	
	FTT absent n=37	FTT present n=13		P-value
Gender				
Female	12 (32.4)	2 (15.4)	14 (28.0 )	0.303
Male	25 (67.6)	11 (84.6)	36 (72.0)	
Total	37 (100)	13 (100)	50 (100)	
Duration of Cast (week)				
Less than equal to 6 times	29 (78.4)	4 (30.8)	33 (66.0)	0.005
More than 6 times	8 (21.6)	9 (69.2)	17 (34.0)	
Total	37 (100)	13 (100)	50 (100)	
Tenotomy				
No	17 (45.9)	4 (30.8)	21 (42.0)	0.340
Yes	20 (4.1)	9 (69.2)	29 (58.0)	
Total	37 (100)	13 (100)	50 (100)	
Laterality				
Unilateral	19 (51.4)	5 (38.5)	24 (48)	0.424
Bilateral	18 (48.6)	8 (61.5)	26 (52)	
Total	37 (100)	13 (100)	50 (100)	

## Discussion

Painless, pliable, and plantigrade foot with limited ankle arthrosis remains the primary objective of treatment of this complex deformity. But even after surgical and non-surgical treatment of CTEV, deformation of the talar dome is observed. There is no consensus to date on the factors that contribute to it. In the long history of treatment of idiopathic congenital clubfoot, various radiological parameters have been suggested to measure the effectiveness of treatment strategies [1]. Most of the studies conducted on these patients are based on clinical scores or mailed questionnaires and, if done, radiological analysis is based on gross ankle arthrosis. Talus shape is perceived to be a key parameter after CTEV treatment, but no rigorous analysis is ever mentioned, including the degree of deformation contributing to severe or non-significant morbidity.

Kolb et al [11] in their study divided talar shape deformation into flat-top talus group and small-dome talus group based on increased radius to length (R/L) or decreased R/L ratios, respectively. They stated that the small-dome talus group has outcomes similar to the normal-dome talus, while the FTT group had significant ankle arthrosis. In their study, moderate to severe deformation of the talus was seen in 44 % of the treated feet. According to Dunn [10], the classification of the flattop talus deformation is based on the morphologic appearance showing flattening and incongruity of the talus bone. Bach et al. [8] also described decreased dynamic range of ankle motion in feet with talar flattening.

Talus deformation was mainly reported following surgical treatment of clubfoot deformity [12], but there are reports on flat-top talus after Ponseti treatment [13]. Sullivan et al. [14] in their study on evaluation of FTT on MRI concluded that FTT incidence is significantly increased if the casting is continued for prolonged periods before surgery. They suggested early surgery if the foot is not corrected after four to six weeks of manipulation and casting. Sinha et al. [15] in their study found only one (01) patient out of 30 having flat-top talus, which could be due to the increased number of casts (n=18), and dorsiflexion achieved was less (3 degrees) even after tenotomy, which is consistent with our study results.

Limitations of our study include smaller sample size, though it is a consecutive series and will continue to add sample as the number of kids completing the treatment will continue, and short-term follow-up as most of the study participants are in their first decade so we do not know the effect of flat-top talus causing significant ankle arthritis leading to morbidity and further surgeries in future. We did not use standard measurements based on the R/L dimensions, and we named the flat-top talus based on maximum talar dome flattening, while mild to moderate deformations were not measured. While two consultants (with more than 10 years experience) checked the results of FTT in our analysis and interobserver variability was not relevant, there is a risk of intra- and inter-observer variability because measurement parameters are not standardized yet, though mose circle method defines sphericity of talar dome as normal, slightly flattened, and greatly altered or flat.

## Conclusions

One of the complications of the Ponseti procedure is the flat-top talus. The shape of the talus not only defines the movement of the ankle but is also a primary predictor of ankle arthrosis onset. Our analysis does not comply with the hypothesis that the talus is crushed against intact tendoachilles (i.e., without tenotomy), but this involves research on a wider population set and longer follow-up.

We recommend examining every child completing casting and bracing procedure to undergo ankle radiograph standing lateral view to assess talus shape and long-term follow-up of patients with clubfoot developing ankle arthritis due to talus shape alteration caused by excessive number of casts.
